# Predicting progression of aortic stenosis by measuring serum calcification propensity

**DOI:** 10.1002/clc.23922

**Published:** 2022-11-03

**Authors:** Reto Kurmann, Eric Buffle, Andreas Pasch, Christian Seiler, Stefano F. de Marchi

**Affiliations:** ^1^ Department of Cardiology University Hospital Bern Freiburgstrasse Bern Switzerland

**Keywords:** aortic sclerosis, aortic stenosis, calcification propensity, echocardiography, valvular heart disease

## Abstract

**Background:**

The aim of this prospective, double‐blinded study in patients with aortic sclerosis was to determine whether a new calcification propensity measure in the serum could predict disease progression.

**Methods:**

We included 129 consecutive patients with aortic sclerosis as assessed during a routine clinical echocardiographic exam. Clinical, echocardiographic, and serum laboratory parameters were collected, including a new blood test providing an overall measure of calcification propensity by monitoring the maturation time of calciprotein particles (T50 test). The echocardiographic exam was repeated after 1 year. Multiple regression analysis was performed to identify independent predictors of the annual increase of peak transvalvular Doppler velocity (∆vmax). Furthermore, the accuracy of the T50 test to detect patients with the most marked stenosis progression was assessed by receiver operating characteristic (ROC)‐analysis.

**Results:**

Mean age was 75 ± 9 years, 79% were men. The T50 was 271 ± 58 min. Overall, there was no significant stenosis progression between baseline and follow‐up (∆vmax 3.8 ± 29.8 cm/s, *p* = ns). The T50 test was not found to be an independent linear predictor in multivariate testing. By ROC‐analysis, however, a T50‐value ≤ 242 min was able to significantly detect a ∆vmax above the 90th percentile (∆vmax ≥ 43 cm/s, AUC = 0.67, *p* = .04, Sensitivity = 69%, Specificity = 70%).

**Conclusions:**

The T50 test showed a modest but significant ability to identify a pronounced aortic stenosis progression in patients with aortic sclerosis. The test could not be established as an independent linear predictor of disease progression, possibly due to the low valvular disease burden and short follow‐up interval.

## 1 INTRODUCTION

Aortic stenosis is the most common valvular heart disease and an important public‐health problem. It is present in approximately 25% of all adults aged above 65 years.^1^ Decisions about surgical or interventional aortic valve replacement are based on symptoms and measures of valvular and ventricular function using echocardiography.^2^, ^3^, ^4^ Such valvular affections are the result of a chronic progressive disease, usually starting with hemodynamically nonsignificant aortic sclerosis, and then progressing to severe stenosis over time. There is no uniform pattern of progression. Instead, marked differences not only between individuals, but also during the time course of the disease can be observed.^5^ Aortic sclerosis progresses to mild aortic stenosis in <15% of patients over 2–7 years.^6^, ^7^ Once moderate stenosis is present (jet velocity >3 m/s), the average progression is 0.3 m/s per year, but still highly variable.^8^, ^9^, ^10^, ^11^ When peak jet velocity exceeds 4 m/s, symptom‐free survival without valve replacement is significantly reduced.^5^, ^12^

In the past, several prospective studies were performed to enhance the predictability of disease behavior. Some determinants of rapid progression and adverse outcome have been identified, such as age, gender, cardiovascular risk factors, B‐natriuretic peptide, stenosis severity, degree of valvular calcification, and others. Although it appears that progression is more rapid in degenerative calcific disease than congenital or rheumatic disease,^10^, ^12^, ^13^, ^14^ predicting progression individually is still prone to large errors even when considering these determinants.^5^ Therefore, regular clinical follow‐up is mandatory in patients with asymptomatic aortic valve affections.^15^ In patients with aortic sclerosis without severe stenosis, it is desirable to find a strong predictor of rapid disease progression. This would allow anticipating cardiovascular deterioration by identifying individuals at particular risk.

From a biochemical and histological point of view, aortic sclerosis is a valvular disease characterized by focal plaque‐like lesions containing microscopic calcifications.^9^, ^16^ Because calcium and phosphate concentrations in serum are near supersaturation, the balance of inhibitors and promoters critically influences the development of calcification. The serum protein fetuin‐A is a major systemic inhibitor of calcification.^17^, ^18^, ^19^ Together with additional blood components, fetuin‐A prevents the supersaturated calcium and phosphate from precipitating by forming soluble colloidal protein–mineral nanoparticles and is therefore an integral part of the defense system preventing calcifications. Low serum concentrations of fetuin‐A are associated with a reduced capacity to inhibit calcification in vitro.^17^, ^20^, ^21^ Calcification takes place when this humoral line of defense is overwhelmed.

Koos et al. showed that serum levels of the calcification inhibitor fetuin‐A are associated with the progression of aortic valve calcifications and major adverse clinical events, independent of the renal function and inflammation.^22^, ^23^

A novel in vitro blood test developed by Pasch et al. provides an overall measure of calcification propensity by monitoring the maturation time (T50) of calciprotein particles. First published clinical data indicate that the T50 test is a helpful biomarker for the prediction of future vascular calcifications.^24^

Fetuin‐A is an important determinant of the T50 test which goes beyond fetuin‐A measurement by functionally integrating the activities of all calcification inhibitors and promotors in the serum into a single readout.

The purpose of this study in patients with aortic sclerosis with and without stenosis is to assess the ability of the T50 test to predict progression of aortic stenosis.

## 2 METHODS

### 2.1 Patient recruitment and study protocol

One hundred twenty‐nine patients referred to our Department of Cardiology for routine echocardiography showing an aortic sclerosis with or without stenosis were prospectively included. Exclusion criteria were: Age <18 years, aortic valve replacement scheduled within 1 year after inclusion, any aortic valvular disease other than degenerative sclerotic, bi‐ or unicuspid valves, known disease with expected survival <1 year, known malignant tumor, subvalvular obstruction (in left ventricular outflow tract) with mean pressure gradient >10 mm Hg. Patients underwent all exams described below during baseline visit, and an additional follow‐up transthoracic echocardiography after 1 year.

The study followed a strict double‐blinded design: the patients and the investigators other than A.P. were blinded for the results of the T50 test. The investigator A.P. (as responsible for the T50 test) was blinded for all clinical and echocardiographic data. Unblinding was performed after completion of all follow‐up examinations.

The study was approved by the Ethics Committee of the Canton of Berne, Switzerland (KEK‐BE: 087/2014).

### 2.2 Patient and public involvment statement

It was not appropriate or possible to involve patients or the public in the design, or conduct, or reporting, or dissemination plans of our research.

### 2.3 Echocardiography

Patients were examined in a left lateral decubitus position using a Philips IE33, Epiq 5, or GE Vingment E95 ultrasonography system. The exam consisted of a comprehensive assessment of cavity and wall dimensions, ventricular and valvular function, morphologic appraisal, and pressure predictions. This procedure was performed in accordance to international guidelines for echocardiography. In particular, aortic stenosis severity was measured using peak aortic jet velocity from using continuous‐wave Doppler tracing, recorded from the window yielding the highest velocity signal.^5^, ^25^ Progression between baseline and follow‐up exam was expressed as peak flow velocity change. To account for some interindividual variations in the follow‐up‐interval, the change in peak flow velocity was normalized to a 12 months interval (∆vmax).

### 2.4 T50 test and other laboratory tests

With the blood samples taken during the baseline visit, a T50 test was performed by Calciscon AG. This test consists of adding calcium and phosphate to serum to trigger the formation of primary calciprotein particles (CPP). As nano‐suspension of calcium‐phosphate, these particles represent a defense mechanism of the serum against calcification. Primary CPPs undergo spontaneous transition to secondary CPPs. The formation of these particles represents calcification. In the T50 test, the time elapsed for the transformation of 50% of the particles is measured optically and is specific for individual sera.^26^

Other laboratory tests included creatinine, albumin, calcium, phosphate, high‐sensitivity C‐reactive protein, LDL cholesterol, venous blood‐gas analysis, brain natriuretic peptide and hemoglobin. Measurements took place at the central laboratory of our clinic. There was no blinding for these laboratory results because the information was used for routine clinical purposes.

### 2.5 Clinical data

Assessment of clinical baseline characteristic included NYHA‐class, angina pectoris CCS‐class, syncope, personal history of coronary artery disease, diabetes mellitus, hypertension, smoking, chronic inflammatory disease and malignancy, family history for cardiovascular disease, and current medication.

### 2.6 Statistical analysis

All patient data was collected in a custom data set in REDCap.^27^ Intraindividual comparisons of continuous variables between baseline and follow‐up were performed using a paired Student's *t* test. To assess the independent predictors of ∆vmax, a two‐step approach was chosen: in the first step, univariate analysis relating all available test variables to ∆vmax was performed to restrict the choice of candidates for further multivariate testing. Univariate testing consisted of linear regression analyses for continuous and unpaired Student's *t* tests for dichotomous, categorical test variables. A threshold of *p* < .10 was used to select candidates for multivariate analysis, consisting of a standard multiple regression model. Dichotomous variables were used as dummy variables in multiple regression analysis. Assumption testing was performed using the following two statistical procedures: (1) A Goldfeld‐Quandt test was used to test the assumption of homoscedasticity of the error with a *p* value < .05 considered as mandatory. (2) Collinearity statistics to exclude multicollinearity between the test variables (a variance inflation factor <10 was defined as mandatory). The results of the overall regression fittings were calculated, including the intercept and standardized coefficients of the multiple regression equation (*β*), adjusted coefficient of determination (adjusted *r* ^2^), and analysis of variance (ANOVA) *F*‐ and *p* values of the overall regression model. Only variables with significant nonzero slopes in the regression equation were considered independent predictors of aortic stenosis progression. These analyses were performed using the R 3.5.2 statistical software.

In addition, patient were assigned to two groups: patients above and below the 90th percentile of ∆vmax. The ability of the T50 test to diagnose the group above the 90th percentile was assessed using a receiver operating characteristic (ROC) analysis. This computation was performed on GraphPad Prism 7 (GraphPad Software).

A *p*‐value < .05 was considered statistically significant in all analyses.

## 3 RESULTS

### 3.1 Baseline characteristic

Baseline characteristics are given in the supplementary material. Mean age was 75 ± 9 years, 79% were male. The T50 was 271 ± 58 min, ranging from 142 to 480 min. The mean follow‐up period was 386 ± 66 days.

### 3.2 Predictors of ∆vmax: Univariate analysis

The univariate relations of the initial test variables with ∆vmax are listed in Table 1 for continuous variables and in Table 2 for categorical variables. Based on the selection criteria described above, the following six variables were chosen for further multivariate testing: age, left ventricular ejection fraction, aspirin, statins, dyslipidemia, family history of coronary artery disease (CAD), and the T50 test. Hematocrit, Base excess, venous PCO_2_ and known coronary artery disease were excluded from further testing due to excess collinearity.

**Table 1:** Results of the simple regression analyses between the continuous explanatory variables and aortic valve velocity progression per year in cm/s

Variable	Intercept	*β*	*r* ^2^	*p* value
T50; min	0.0	1.3	.02	.84*
Calcium; mmol/L	−26.0	64.3	.11	.20
Phosphate; mmol/L	−8.0	11.8	.06	.50
Magnesium; mmol/L	2.3	1.9	.01	.92
Creatinine; mcmol/L	0.0	1.9	.06	.53
Albumin; g/L	−1.0	39.3	.11	.24
BNP; pg/ml	0.0	4.3	.03	.75
hs‐CRP; mg/L	0.5	2.3	.08	.40
LDL; mmol/L	3.3	−4.5	.10	.26
Hb; g/L	0.0	3.6	.01	.99
Thrombocytes; G/L	0.0	4.2	.01	.96
Leukocytes; G/L	−0.9	10.1	.06	.50
pH	−46.4	346.3	.07	.45
HCO_3_; mmol/l	0.4	−7.0	.04	.65
PO_2_ venous; mm Hg	−0.1	7.8	.06	.53
P50 venous; mm Hg	0.9	−20.3	.05	.57
Left ventricular mass; g	0.1	−6.4	.12	.16
LV ejection fraction; %	−0.4	28.4	.16	.07*
Mean blood pressure; mm Hg	0.0	1.4	.01	.89
Body mass index; kg/m^2^	−0.9	28.2	.13	.13
Heart rate;/min	−0.1	8.7	.03	.74
Age; year	0.5	−35.6	.16	.07*

Abbreviations: BNP, brain natriuretic peptide; CRP, C‐reactive protein; LDL, low‐density lipoprotein.

* Variables selected for the multiple regression analysis. Variables deliberately not tested due to collinearity: Hematocrit, base excess, PCO_2_ venous.

**Table 2:** Independent group comparison of aortic valve velocity progression per year means in cm/s between different categorical test variables

Variable	*n* (yes/no)	Yes	No	*p* value
Aspirin	77/52	−1 ± 30	11 ± 29	.02*
Vitamin K antagonists	22/107	13 ± 27	2 ± 30	.10
NOAC	7/122	4 ± 30	4 ± 30	.99
P2Y12 inhibitors	22/107	12 ± 27	2 ± 30	.14
Beta‐blockers	77/52	5 ± 31	2 ± 28	.49
ACE inhibitors	49/80	2 ± 33	5 ± 28	.70
Sartans	55/74	4 ± 28	4 ± 31	.94
Calcium channel blockers	44/85	5 ± 33	3 ± 28	.84
Statins	93/36	0 ± 29	15 ± 29	.01*
Loop diuretics	24/105	7 ± 28	3 ± 30	.53
Thiazid diuretics	31/98	5 ± 33	3 ± 29	.84
Aldosterone antagonists	9/120	2 ± 18	4 ± 30	.82
Vitamin D supplementation	38/91	4 ±± 28	4 ± 31	.93
Calcium supplementation	27/102	8 ± 32	3 ± 29	.39
Male gender	98/31	5 ± 30	−1 ± 29	.26
Chronic inflammatory disease	17/112	−1 ± 35	5 ± 29	.51
Immunosuppressive treatment	26/103	−3 ± 34	5 ± 29	.28
Hypertension	116/13	3 ± 30	7 ± 33	.67
Active smoker	10/119	−7 ± 31	5 ± 30	.28
Dyslipidemia	96/33	1 ± 28	12 ± 33	.09*
Diabetes mellitus	34/95	−2 ± 30	6 ± 30	.21
Family history of CAD	35/94	−5 ± 27	7 ± 30	.03

Abbreviations: ACE, angiotensin‐converting enzyme; AVVPY, aortic valve velocity progression per year; CAD, coronary artery disease; NOAC, non‐vitamin K antagonist oral anticoagulants.

* Variables selected for the multiple regression analysis. Variables deliberately not tested due to collinearity: Known coronary artery disease.

### 3.3 Predictors of ∆vmax: Multivariate analysis

The results of the multiple regression analysis are listed in Tables 3 and 4. A mildly determined, but statistically significant overall model was found (*F* = 3.03, *p* value = .01, adjusted *r* ^2^ = .10). Among the test variables entered in the model, only Aspirin intake was significantly related to ∆vmax (*β* = −12.3, *p* = .02), with intake being associated with lower ∆vmax. All other test variables, including T50, were not significantly related to ∆vmax.

**Table 3:** Results of the multiple regression analysis with selected test variables

Variable	β	*p* value
T50; min	.0	.68
LV ejection fraction; %	−.4	.12
Age; year	.3	.36
Aspirin	−12.3	.02
Statins	−10.6	.1
Dyslipidemia	−4.7	.46
Family history of CAD	−8.5	.16

Abbreviations: CAD, coronary artery disease; LV, left ventricular.

**Table 4:** Global results of the multiple regression analysis

Tests names	Results
Tests	
Adjusted *r^2^*	.10
*F* statistics	3.03
*p* value	.01
Number of patients	129

### 3.4 ROC analysis

**Figure 1:**
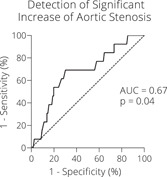
ROC analysis for the detection of a significant increase in aortic stenosis. ROC, receiver operating characteristic.

Fourteen patients with a ∆vmax value above the upper 90th percentile were depicted. The mean ∆vmax in this subgroup was 58 cm/s (range 43–91 cm/s), and T50 was 241 ± 48 min. In the other group, mean ∆vmax did not significantly differ from zero (range −66 to 42 cm/s), and T50 was 274 ± 59 min. The ROC analysis is shown in Figure 1. A T50 value ≤242 min was able to detect the patients whose ∆vmax was above the 90th percentile with a sensitivity of 69% and a specificity of 70%. The overall ROC analysis was statistically significant (*p* = .04) with an area under the curve (AUC) of 0.67.

## 4 DISCUSSION

This prospective, double‐blinded observational study in 129 patients with aortic sclerosis shows that measuring individual serum calcification propensity (as assessed by the T50 test) allows identifying patients with the most pronounced progression of aortic stenosis in advance. The diagnostic accuracy is modest, but statistically significant. We found no linear relation between T50 and stenosis progression.

The T50 test has previously been established as predictor of morbidity and mortality, primarily by investigating patient populations with vascular calcification. In 6231 individuals who participated in the PREVEND‐Study in Groningen, Netherlands, Eeldering et al. found an association between T50 and cardiovascular mortality.^28^ Smith et al. showed that in patients with chronic kidney disease, T50 is associated with an increase of aortic stiffness over a period of 5 years, the finding of which was potentially related to the increased mortality found in the study.^24^

Fewer data exist on the role of serum‐driven calcification in degenerative aortic valve stenosis. In an in‐vitro study, Rattazzi et al. showed that inhibitors of tissue calcification, such as pyrophosphate, are significantly reduced in calcified aortic valve tissue,^29^ encouraging the conclusion that a test capable of measuring serum‐driven calcification could predict degenerative valve progression. In our study, however, multiple regression analysis (involving clinical, echocardiographic, and laboratory parameters) could not establish the T50 test as an independent predictor of disease progression over 12 months. The available data does not offer a direct explanation for the lack of such a relation, but we suspect it to be mainly attributable to the short‐term follow‐up period and to the low average valvular disease burden. This is in line with previous studies on the natural course of aortic stenosis progression showing that in patients with moderate disease, significant progression requires several years to occur.^7^ It is also possible that a certain threshold of serum calcification propensity must be exceeded for pronounced calcification to occur, since our subgroup with a pronounced disease progression had a significantly lower T50 time. It can be assumed that serum‐driven calcification processes are likely involved in the pathogenesis of calcific aortic valve disease.

Some other predictors of stenosis progression have been identified in previous studies, that is, jet velocity at baseline,^5^ the Agatson score^7^ or diabetes.^13^ The former shows the nonlinearity of disease progression, i.e. accelerating dynamics with advancing severity. The main difference to our study population consists not only of the sample size, but also of the more severe stenosis in the previous studies. Despite this, the T50 test was capable of identifying the subgroup with particularly pronounced disease progression with a single visit. This is of particular interest, because such patients may require more frequent follow‐up exams even in the presence of mild to moderate stenosis.^12^ A 1‐year vmax progression ≥ 22 cm/s has been associated with a poorer prognosis.^13^ In our study, a T50 below 242 min identified patients with an annual vmax progression of at least 43 cm/s, thus indicating a potentially important impact on prognostic patient stratification.

Aspirin and statins were both inversely related to disease progression in univariate analysis, whereas only Aspirin emerged as an independent predictor in multivariate analysis, that is, intake being related to less progression. While similar results regarding dyslipidemia have been found previously,^13^ Aspirin has not emerged as inhibitor of calcification in other studies. It remains unclear whether this finding is of pathophysiological importance.

The proportion of patients with chronic kidney disease and kidney transplant is relatively high in this study population, reflecting the referral practice in our tertiary center. These patients are known to have a pronounced serum calcification propensity and reduced T50 Therefore, these patients may have contributed to a larger than normal range of T50‐values.^30^ A comparison with the T50‐values in a general population of the PREVEND‐study, however, strongly suggests a very similar distribution.^28^

In conclusion, serum calcification propensity seems involved in aortic stenosis progression, with the T50 test capable of identifying individuals with the most pronounced stenosis progression with moderate diagnostic performance. A larger study with longer follow‐up interval is needed to elucidate whether the T50 test is of prognostic relevance in aortic sclerosis patients.

## AUTHOR CONTRIBUTIONS

The authors have respectively contributed to the following task of the article: Reto Kurmann: Conduct. Eric Buffle: Reporting. Andreas Pasch: Conduct. Christian Seiler: Reporting. Stefano F. de Marchi: Planning, conduct, and reporting.

## CONFLICTS OF INTEREST

AP is an employee and stockholder in Calciscon AG, Nidau, Switzerland, a company that commercializes the T50 test. The authors declare no conflicts of interest.

## Supporting information

Supplementary information.

## Data Availability

The data that support the findings of this study are available from the corresponding author, [SdM], upon reasonable request.
