# Supplementary material for: Predicting progression of aortic stenosis by measuring serum calcification propensity
Source: Clin Cardiol. 2022 Nov 3;45(12):1297–302. doi: 10.1002/clc.23922 (PMC9748749; doi:10.1002/clc.23922)
Supplement: Supplementary file 1 — Supplementary information. [file CLC-45-1297-s001.docx]

| Supplementary Material: Patients characteristics | |
| --- | --- |
| Clinical and haemodynamical findings  Age; yr | 75±9 |
| Body mass index; kg/m2 | 26.8±4.3 |
| Heart rate; /min | 70±13 |
| Left ventricular mass; g | 198±72 |
| LV ejection fraction; % | 56±11 |
| Mean blood pressure; mmHg | 97±15 |
| Laboratory tests  Calcium; mmol/l | 2.33±0.13 |
| Phosphate; mmol/l | 1.00±0.22 |
| Magnesium; mmol/l | 0.81±0.12 |
| Creatinine; mcmol/l | 124±110 |
| Albumin; g/l | 37±3 |
| BNP; pg/mL | 186.03±324.4 |
| hs-CRP; mg/L | 3.1±4.7 |
| LDL; mmol/l | 2.48±0.89 |
| Hb; g/l | 134±19 |
| Haematocrit; % | 40±5 |
| Thrombocytes; G/l | 228±64 |
| Leucocytes; G/l | 7±2 |
| pH | 7.38±0.04 |
| PCO2 venous; mmHg | 43±6 |
| HCO3; mmol/l | 245±4 |
| Base excess; mmol/l | 0.08±2.69 |
| PO2 venous; mmHg | 39±16 |
| P50 venous; mmHg | 28±2 |
| Medications  Aspirin | 77 (60%) |
| Vitamin K antagonists | 22 (17%) |
| NOAC | 7 (5%) |
| P2Y12 inhibitors | 22 (17%) |
| Beta-blockers | 77 (60%) |
| ACE inhibitors | 49 (38%) |
| Sartans | 55 (43%) |
| Calcium channel blockers | 44 (34%) |
| Statins | 93 (72%) |
| Loop diuretics | 24 (19%) |
| Thiazid diuretics | 31 (24%) |
| Aldosterone antagonists | 9 (7%) |
| Vitamin D supplementation | 38 (29%) |
| Calcium supplementation | 27 (21%) |
| Cardiovascular risk factors  Male gender | 98 (76%) |
| Chronic inflammatory disease | 17 (13%) |
| Immunosuppressive treatment | 26 (20%) |
| Hypertension | 116 (90%) |
| Active smoker | 10 (8%) |
| Dyslipidemia | 96 (74%) |
| Diabetes mellitus | 34 (26%) |
| Known CAD | 34 (26%) |
| Family history of CAD | 35 (27%) |
| Study specific measurements  Progression of AV velocity per year; cm/(s · yr) | 3.8±29.8 |
| Time to follow-up; days | 386±66 |
| T50; min | 271±58 |
| Metric variables are given as means ± standard deviation. Categorical variables are given as numbers. Abbreviations: ACE: Angiotensin-converting enzyme. BNP: Brain natriuretic peptide. CAD: Coronary artery disease. CRP: C-reactive protein, LDL: Low-density lipoprotein, NOAC: Non-vitamin K antagonist oral anticoagulants. | |
